# Compassion for Others and Self-Compassion: Levels, Correlates, and Relationship with Psychological Well-being

**DOI:** 10.1007/s12671-017-0777-z

**Published:** 2017-07-18

**Authors:** Angélica López, Robbert Sanderman, Adelita V. Ranchor, Maya J. Schroevers

**Affiliations:** 1 Department of Health Psychology, University Medical Center Groningen, University of Groningen, Hanzeplein 1, De Brug FA12, 9700 RB Groningen, The Netherlands; 2 Department of Psychology, Health and Technology, University of Twente, Enschede, The Netherlands

**Keywords:** Compassion for others, Self-compassion, Depressive symptoms, Negative affect, Positive affect, Demographics

## Abstract

Compassion for others and self-compassion are assumed to be closely related concepts. Yet, as they have been mostly studied separately, little is known about their relationship and to what extent they differ or resemble each other with respect to their correlates. This cross-sectional study aimed to gain knowledge on their mean levels, interrelationship, and relationships to psychological well-being and demographic factors. A community sample of 328 adults completed a series of standardized self-report questionnaires to assess compassion for others, self-compassion, depressive symptoms, negative affect, and positive affect. Results showed that compassion for others and self-compassion were not significantly related. Self-compassion was more strongly related to negative and positive indicators of affect than compassion for others. Compassion for others was higher in women than in men, and in low educated individuals compared to higher educated individuals. In contrast, self-compassion was lower in low educated individuals. Future research can build up on these findings to enlarge the understanding of how compassion for others and self-compassion relate and differ from each other.

## Introduction

The interest on the benefits of compassion for others and self-compassion has grown rapidly during the last decade. Although these two concepts are assumed to be closely related, research examining their association is notoriously scarce. There is compelling evidence suggesting that compassion for others is a distinct emotion rooted in evolution (Goetz et al. 2010). Specifically, it is suggested that compassion for others evolved as part of a caregiving response to vulnerable offspring, that it promotes cooperative relations between nonkin, and that compassionate mates are preferred. According to this approach, compassion for others is the emotion that arises when witnessing another’s suffering and that subsequently motivates a desire to help. Compassion for others can be understood as a state or as a trait (Goetz et al. 2010). The state consists in a brief and context-related emotional displayed of compassion, triggered by a clear cause. The trait involves the tendency to experience compassion across different situations, that is, a general style of emotional response that is transversal to time and context.

The most commonly used conceptualization of self-compassion was proposed by Neff (2003b). Neff (2003b) conceptualizes self-compassion as an attitude that is relevant to every personal experience of suffering and that entails three interacting components: (1) self-kindness vs self-judgment, (2) a sense of common humanity vs isolation, and (3) mindfulness vs over-identification (Neff 2003a). Self-kindness denotes treating oneself with tenderness, warmness, and understanding in the face of suffering rather than with harshness and self-judgment. A sense of common humanity refers to seeing one’s failures and painful experiences as part of the large human condition rather than feeling isolated and cut-off of the rest of humanity. The mindfulness component involves maintaining a balanced awareness of the painful experiences instead of over-identifying with painful thoughts and emotions.

Only few studies have explored the relationship between compassion for others and self-compassion. In a fMRI study, it was found that self-compassion engages similar brain regions as expressing compassion towards others (Longe et al. 2010; Lutz et al. 2008). Another study found small positive correlations between compassion for others and self-compassion in community adults and meditators, with a somewhat stronger association for the meditators’ group (Neff and Pommier 2012). In line, in a series of four experiments, it was found that activating support-giving schemas increased self-compassion (Breines and Chen 2013). Finally, and contrasting the previously mentioned evidence, in an experimental study, it was found that individuals with low and high self-compassion did not differ in their evaluations of others (Leary et al. 2007).

Compassion for others and self-compassion seem to be beneficial for individuals’ psychological well-being. The evidence for the association of compassion for others and well-being comes primarily from experimental and intervention studies. For instance, after a brief compassion training in a sample of healthy adults, participants’ experiences of positive affectivity were higher compared to a control condition (Klimecki et al. 2012). In addition, community adults that performed a daily compassionate action towards others in a 1-week task study showed increases in self-reported happiness at the end of the week, compared to a control condition (Mongrain et al. 2011). The relationship between self-compassion and psychological well-being has been largely explored through survey methods. According to a systematic review, high self-compassion is associated with reduced stress, anxiety, and depressive symptoms (MacBeth and Gumley 2012). Self-compassion also relates with improvements in self-reported indicators of positive affectivity, such as greater happiness, optimism, positive affect, and life satisfaction (Neff 2003a; Neff et al. 2007).

So far, the limited number of studies examining compassion for others together with self-compassion suggest that they involve similar brain regions (Longe et al. 2010) and that those individuals who are more compassionate towards others could be more compassionate towards themselves (Breines and Chen 2013; Neff and Pommier 2012). However, there is still a limited understanding of how much these concepts are similar or different from each other; specifically, descriptive data is missing.

This cross-sectional study among community adults aims to give insight into the mean levels of compassion for others and self-compassion, their association, and their relationship with psychological well-being (i.e., depressive symptoms, negative affect, and positive affect) and demographic factors. We measured compassion for others according to the conceptualization of Goetz et al. (2010) and self-compassion according to the conceptualization of Neff (2003b). Recent evidence suggests that the positive (i.e., self-kindness, common humanity, and mindfulness) and negative (i.e., self-judgment, isolation, and over-identification) components of Neff’s conceptualization measure distinct constructs and can better be used separately as measures of self-compassion and self-coldness, respectively (Costa et al. 2015; López et al. 2015; Muris et al. 2016). In this study, we present results for self-compassion and self-coldness though we focused on those of self-compassion since our main interest was to assess the positive experience of self-compassion in relationship with compassion for others. Based on previous research, we expected a small significant association between compassion for others and self-compassion (Breines and Chen 2013; Neff and Pommier 2012). In addition, we expected self-compassion to be significantly related to depressive symptoms, negative affect, and positive affect (MacBeth and Gumley 2012; Neff 2003a; Neff et al. 2007), and compassion for others to be significantly related to positive affect (Jazaieri et al. 2014; Mongrain et al. 2011).

## Method

### Participants

The study was conducted among 328 individuals from the general population. The sample included 181 females (55.2%) and 147 males (44.8%). Mean age was 57 years (*SD* = 15.2). The complete demographic characteristics of the sample are presented in Table 1.

**Table 1:** Participants’ demographic characteristics and means (*SD*) of all study variables

	Study sample (*N* = 328)
Mean age in years (*SD*)	57 (15.2)
Gender (% female)	55.2
Marital status (%)	
Married/cohabiting	78.8
Single	6.4
Widowed	8.9
Divorced	3.4
Other	2.4
Education (%)	
Low	17.1
Middle	51.6
High	31.2
Working status (%)	
Employed	49.2
Retired	22.6
Housework	8.2
Volunteer	5.0
Disability	5.3
Others	9.4
Presence of physical disease	
No	44.5
One	23.5
Two or more	24.1
Mean of study variables (SD)	
Compassion for others	28.11 (4.29)
Self-compassion	36.87 (7.60)
Self-coldness	27.17 (8.47)
Depressive symptoms	8.65 (8.87)
Negative affect	15.26 (5.71)
Positive affect	30.57 (6.93)

### Procedure

Data was collected as part of a follow-up assessment of a larger study on mindfulness, self-compassion, and quality of life. The community sample was selected from the register offices of five middle size cities in the Netherlands. The follow-up assessment was made in two waves (hereby following the same procedure of the baseline assessment). This study focuses on the data collected during the second wave (450 individuals approached). A questionnaire package was sent to the participants’ home addresses with a return envelope, so they could return the questionnaire without any cost. The complete questionnaire package was in Dutch (the native language of the participants); therefore, the Dutch translations of the self-report scales were used. Data was obtained from 328 participants (73%). We compared the sample of this study with the rest of the follow-up sample and found that the two samples did not significantly differ in age, gender, marital status, education, working status, or presence of physical disease, and either in depressive symptoms, negative affect, or positive affect.

### Measures

#### Compassion for Others

Compassion for others was assessed with the compassion subscale of the Dispositional Positive Emotions Scale (DPES-comp; Shiota et al. 2006). This 5-item subscale assesses the tendency to feel compassion towards people in general using a 7-point Likert scale, with 1 indicating *strongly disagree* and 7 indicating *strongly agree* (e.g., ‘When I see someone hurt or in need, I feel a powerful urge to take care of them’). Total scores can range from 5 to 35, with higher scores indicating greater levels of compassion for others. The DPES-comp has demonstrated a good internal consistency and adequate validity (Shiota et al. 2006). In this study, the DPES-comp had good internal consistency (*α* = .84).

#### Self-Compassion

Self-compassion was measured with the 12 positive items and self-coldness with the 12 negative items of the Self-Compassion Scale (SCS; Neff 2003a). Neff and Vonk (2009) translated the original SCS into Dutch removing 2 of the 26 items from the original English version due to difficulties in translation; thus, the Dutch SCS contains 24 items. The SCS’s 12 positively formulated items measure self-kindness, a sense of common humanity and a mindful approach to suffering (e.g., ‘I am kind to myself when I am experiencing suffering’), and the 12 negatively formulated items measure self-judgment, isolation and over-identification (e.g., ‘I am intolerant and impatient towards those aspects of my personality I don’t like’). The items can be rated on a 5-point Likert scale with 1 indicating *almost never* and 5 indicating *almost always*. In the present study, the sum score of the positive formulated items was used as a measure of self-compassion, and the sum score of the negatively formulated items as a measure of self-coldness. The score of these scales can range from 12 to 60. The internal consistency was good for both self-compassion (*α* = .87) and self-coldness (*α* = .89).

#### Depressive Symptoms

Depressive symptoms were assessed with the Center of Epidemiologic Studies Depression Scale (CES-D; Bouma et al. 1995; Radloff 1977)*.* The CES-D is a 20-item self-report instrument designed to measure current levels of depressive symptomatology in the general population (e.g., ‘I felt depressed’). On a 4-point Likert scale, participants specified the frequency by which each symptom was experienced during the last week (0 indicating *rarely or none of the time* and 3 indicating *most of the time*). After reversing the positively formulated items, a total score can be calculated based on all 20 items. Total scores can range from 0 to 60. Higher scores indicate more depressive symptoms. In this study, the scale showed good internal consistency (*α* = .91).

#### Negative and Positive Affect

Negative and positive affect were measured with the 20-item Positive and Negative Affect Schedule (PANAS; Peeters et al. 1999; Watson et al. 1988). This instrument is divided into two 10-item scales that assess subjective distress and unpleasant engagement (i.e., negative affect) and feelings of activeness, enthusiasm, and alertness (i.e., positive affect). Participants were asked to rate the extent to which they experienced each particular state during the last week using a 5-point Likert scale (1 indicating *very slightly or not at all* and 5 indicating *very much*). Higher scores in the two scales indicate more negative and positive affect. In this study, the PANAS demonstrated high internal consistency for the negative affect (*α* = .88) and positive affect (*α* = .88) scales.

### Data Analyses

Statistical analyses were conducted in SPSS, 20.0. The association between compassion for others, self-compassion, and self-coldness was tested with Pearson correlation. Next, to examine whether compassion for others, self-compassion, and self-coldness varied according demographic factors (i.e., age, gender, marital status, education, working status and presence of physical disease), several t-tests and ANOVAs were performed testing for differences in the DPES-comp and SCS scores. For these analyses, the variable marital status was dichotomized into married/cohabitating and others, and the variable working status was categorized into employed, retired, and others. Lastly, correlation analyses were conducted to explore the relationships between compassion for others, self-compassion, and self-coldness with depressive symptoms, negative affect, and positive affect. The demographic characteristics of the participants, as well as the means (*SD*) of all study variables, are presented in Table 1.

## Results

The mean level of participants’ compassion for others was *M* = 5.62, that is, in between categories 5–6 (*somewhat agree*–*agree*) of a 7-point Likert scale. The mean level of participants’ self-compassion was *M* = 3.07, that is, close to category 3 (*sometimes*) of a 5-point Likert scale. The mean level of participants’ self-coldness was *M* = 2.26, that is, in between categories 2–3 (*rarely–sometimes*) of a 5-point Likert scale. Compassion for others and self-compassion were weakly, not significantly related (*r* = .10, *p* = .071). Self-coldness had a weak negative significant correlation with self-compassion (*r* = −.18, *p* < .001) and a non-significant association with compassion for others.

Compassion for others was not significantly related to depressive symptoms neither to negative or positive affect. Self-compassion had significant, weak to moderate, negative associations to depressive symptoms and negative affect, and a significant moderate positive association to positive affect (*p* < .001). Self-coldness had a strong positive correlation with depressive symptoms and negative affect, and a weak negative correlation with positive affect (*p* < .001). (Table 2).

**Table 2:** Correlations of compassion for others, self-compassion, and self-coldness with measures of psychological well-being

	Depressive symptoms	Negative affect	Positive affect
Compassion for others	−.001	−.050	.072
Self-compassion	−.318^***^	−.221^***^	.351^***^
Self-coldness	.543^***^	.527^***^	−.240^***^

^***^ *p* < .001

Women showed higher levels of compassion for others than men (*t*(326) = −2.21, *p* = .028). In addition, compassion for others was higher in low educated individuals, compared to middle- and high-educated individuals (*F*(2, 324) = 4.90, *p* = .008). Age was weakly positively correlated with compassion for others (*p* < .05). Low-educated individuals had lower levels of self-compassion than middle- and high-educated individuals, and middle-educated individuals had lower levels of self-compassion than high-educated individuals (*F*(2, 323) = 12.34, *p* < .001). Women showed higher levels of self-coldness than men (*t*(326) = −2.92, *p* = .004). In addition, self-coldness was lower in retired individuals, compared to those with a pay work or doing other activities (*F*(2, 315) = 4.60, *p* = .011). Age was weakly negatively correlated with self-coldness (*p* < .05) (Table 3).

**Table 3:** Means (*SD*) of compassion for others, self-compassion and self-coldness for different demographic groups

	Compassion for others (range 5–35)	Self-compassion (range 12–60)	Self-coldness (range 12–60)
Age	.116^*^	−.022	−.163^**^
Gender			
Women	27.53 (4.08)^a*^	37.29 (7.34)	28.38 (8.90)^d**^
Men	28.58 (4.42)	36.34 (7.91)	25.67 (7.66)
Marital status			
Married/cohabiting	28.30 (4.31)	37.11 (7.33)	27.07 (8.19)
Others	27.39 (4.23)	35.92 (8.62)	27.40 (9.53)
Education			
Low	29.73 (3.98)^b**^	33.11 (7.60)^c**^	27.68 (8.61)
Middle	27.81 (4.16)	36.67 (7.66)	27.67 (8.66)
High	27.73 (4.52)	39.17 (6.61)	26.08 (8.08)
Working status			
Employed	27.67 (4.57)	36.90 (7.04)	27.87 (9.33)
Retired	28.18 (4.01)	35.85 (7.52)	24.46 (6.61)^e**^
Others	28.79 (4.05)	37.72 (8.52)	27.98 (8.23)
Presence of physical disease			
No	27.56 (3.93)	37.43 (7.69)	26.31 (8.26)
One	28.14 (4.95)	36.89 (7.28)	28.09 (8.66)
Two or more	28.57 (4.19)	36.38 (7.86)	28.60 (8.95)

^a^Significant difference in compassion for others between women and men

^b^Significant differences in compassion for others between low educated and middle/high educated

^c^Significant differences in self-compassion between low educated and middle/high educated, and between middle educated and high educated

^d^Significant difference in self-coldness between women and men

^e^Significant differences in self-coldness between retired and employed/others

^**^ *p* < .01, ^*^ *p* < .05

## Discussion

This study aimed to explore the mean levels of compassion for others and self-compassion in the general population, their interrelationship, and their association to psychological well-being and demographic factors. Results showed that compassion for others and self-compassion were not significantly related. Self-compassion was associated with lower levels of depressive symptoms and negative affect, and higher levels of positive affectivity, while compassion for others was not significantly related to psychological well-being. Women and lower-educated individuals reported to be more compassionate for others than their counterparts. Lower-educated individuals reported less self-compassion than higher-educated individuals.

The mean levels of compassion for others and self-compassion observed in this study are similar to those reported in previous studies (Costa et al. 2015; Körner et al. 2015; Oveis et al. 2010; Stellar et al. 2012). The finding that on average, participants tend to feel compassion towards others is in line with the notion that compassion is a distinct emotion that denotes important evolutionary purposes (Goetz et al. 2010). The finding that on average, participants reported to experience self-compassion only sometimes might be explained by an inherent difficulty of expressing compassion towards oneself (Gilbert et al. 2011). In a qualitative study, individuals with depression reported that being self-compassionate seemed difficult and challenging (Pauley and McPherson 2010). This might also be the case for non-depressed individuals. Gilbert et al. (2011) argued that highly self-critical people could experience a fear to be self-compassionate and have difficulties in developing self-compassion. An alternative explanation for our findings can be that it is socially desirable to report compassion for others more so than for oneself. Our participants belong to a western culture in which positive evaluations by others are highly valued. It would be important to explore the association of social desirability with compassion for others and self-compassion across different cultures.

Interestingly, compassion for others and self-compassion were not significantly associated. Other researchers have found that compassion for others is weakly or not related to self-compassion (Gilbert 2016; Neff and Pommier 2012). This suggests that it is possible to be compassionate towards others but not towards the self, or vice versa. A main difference is that compassion for others seems to have evolved as a desired trait for mate selection, and thus, has important social purposes (Goetz et al. 2010). Self-compassion, in contrast, seems to require of a more advanced cognitive processing and it is limited to the individual. These constructs also differ in the way they are conceptualized and measured, and in turn, this might affect their association. Compassion for others is typically assessed as a one-dimension construct (Shiota et al. 2006), whereas self-compassion is commonly measured as a multi-dimensional construct (Neff 2003b). Currently, a set of three scales is being developed to measure compassion to others, to the self, and from others, with the same items (Gilbert 2016). It would be meaningful to explore the association of self- and other-compassion using this upcoming scale.

Self-compassion showed to be related to negative and positive affective states. Dundas et al. (2015) suggested that self-compassion might relate to lower depressive symptoms by protecting against the increase of self-judging responses. The influence of self-compassion on positive affect can be due to a positive affective response (e.g., experiencing warmth, understanding, and reassurance) in the face of personal distress (Neff 2003b). Complementarily, during less threatening situations, self-compassion can have a resilient effect by promoting healthy behaviors aimed to maintained well-being (Neff 2003b). In contrast, compassion for others did not appear to be significantly related to depressive symptoms, negative affect nor positive affect. Partly in line with our results, two previous studies found that after 1 week of performing daily compassionate acts towards others (Mongrain et al. 2011) and after a 9-week compassion intervention (Jazaieri et al. 2014), participants did not show greater decreases of depressive symptoms compared to a control group, though they did show higher increases of happiness. More research, particularly survey studies, is needed to increase the understanding of how compassion for others relates to psychological well-being.

When exploring compassion for others and self-compassion among different demographic groups, results showed that women reported higher compassion for others compared to men. Past literature has observed this same gender difference in undergraduate students, community adults, and meditators (Neff and Pommier 2012; Stellar et al. 2012). Sprecher et al. (2007) found that women, at a greater degree than men, expect enhanced positive mood as a result of compassionate acts and theorized that it can be due to differences in social role experiences. In line with this, the social role theory of helping (Eagly and Crowley 1986) suggests that gender roles encourage males to perform heroic actions while females to be nurturing and caring. We found no gender differences in self-compassion, similarly to results from a study among undergraduates, community adults, and meditators (Neff and Pommier 2012). A recent meta-analysis, however, found slightly lower self-compassion in women compared to men, as measured by the SCS total score (Yarnell et al. 2015). Another study that explored gender differences across the SCS’s subscales found that women reported significantly higher self-judgment, isolation, and over-identification, and lower mindfulness, compared to men. It could be that the negative aspects of the SCS mainly account for the gender differences in self-compassion, and that when focusing on the positive experience of self-compassion, no significant gender differences emerge.

Low-educated individuals reported higher compassion for others compared to their counterparts. Similarly, previous research found that lower-class individuals reported greater compassion for others during laboratory inductions and real social interactions, compared to upper-class individuals (Stellar et al. 2012). The association between social class and compassion for others was mediated by the perception of distress in others, supporting the idea that lower-class individuals, who often live in more threatening environments, initiate cooperative relationships as a strategy to deal with external threats (i.e., tend-and-befriend response strategy). Self-compassion was found to be lower in lower-educated individuals. It is possible that lower-educated individuals have difficulty in understanding the scale items, and in turn, this affects their scores. Considering that the SCS was pilot tested and validated with university samples (Neff 2003a), some of its items can indeed be complex. More research is needed in order to clarify whether and how education influences self-compassion.

### Limitations

The community sample with equivalent gender distributions and broad age range increases the generalizability of our results. Though, some limitations should be considered when interpreting our findings. Our study is cross-sectional and therefore conclusions regarding causality of self-compassion on psychological well-being cannot be drawn. Another limitation is the dropout of participants between the baseline and follow-up study. Low response rates are not uncommon for mail surveys (Van Horn et al. 2008), and it is possible that the topic of the study and the length of the questionnaire package contributed to the reduction on the response rate. Although the follow-up sample did not significantly differ from the non-respondent sample in age or gender distributions, we did find that higher educated people and individuals married or with a partner were more likely to participate in the follow-up. Finally, it would be important to further examine the association of compassion for others and self-compassion across different cultures and in younger populations (although our sample age ranges from 21 to 91 years old, the 75% is above 40 years old). Future research can build up on the findings from this study to enlarge the understanding of how compassion for others and self-compassion relate and differ from each other.
